# An overview of long non-coding RNAs in ovarian cancers

**DOI:** 10.18632/oncotarget.8089

**Published:** 2016-03-15

**Authors:** Matthieu Meryet-Figuière, Bernard Lambert, Pascal Gauduchon, Nicolas Vigneron, Emilie Brotin, Laurent Poulain, Christophe Denoyelle

**Affiliations:** ^1^ Inserm U1199, Biology and Innovative Therapeutics for Locally Aggressive Cancer (BioTICLA) Unit, Caen, France; ^2^ Normandie University, Caen, France; ^3^ UNICAEN, Caen, France; ^4^ Comprehensive Cancer Center CLCC François Baclesse, Unicancer, Caen, France; ^5^ CNRS, Paris, France

**Keywords:** IncRNA, ovarian cancer

## Abstract

As with miRNAs a decade ago, the scientific community recently understood that lncRNAs represent a new layer of complexity in the regulation of gene expression. Although only a subset of lncRNAs has been functionally characterized, it is clear that they are deeply involved in the most critical physiological and pathological biological processes. This review shows that in ovarian carcinoma, data already available testify to the importance of lncRNAs and that the demonstration of an ever-growing role of lncRNAs in the biology of this malignancy can be expected from future studies. We also underline the importance of their relationship with associated protein partners and miRNAs. Together, the available information suggests that the emerging field of lncRNAs will pave the way for a better understanding of ovarian cancer biology and might lead to the development of innovative therapeutic approaches. Moreover, lncRNAs expression signatures either alone or in combination with other types of markers (miRNAs, mRNAs, proteins) could prove useful to predict outcome or treatment follow-up in order to improve the therapeutic care of ovarian carcinoma patients.

## INTRODUCTION

Ovarian cancer is the leading cause of death from gynecological malignancies worldwide. More than 230,000 new cases are diagnosed each year, at a late dissemination stage in most cases, leading to the death of about 140,000 women [1]. The overall 5-year survival rate is particularly low for the advanced stages. Two main factors may explain this poor prognosis [2]: (i) the asymptomatic nature of this disease during the early stages generally leads to a late diagnosis at a time when it has already spread to the entire peritoneal cavity; (ii) the resistance to conventional carboplatin/taxol chemotherapy. Whereas most patients initially present a positive response to chemotherapy, most of them will relapse and develop a resistance to treatment, leading to a therapeutic dead-end. To date, many efforts have been made to overcome this resistance and to develop targeted therapies specifically dedicated to the treatment of patients with identified genetic alterations, but therapeutic care for ovarian cancers is lagging behind other cancer types in which such therapies have already been incorporated into standard treatment. As a consequence, the management of ovarian cancer has not improved in recent decades, underlining the need for better understanding of its biology.

The identification of the roles and functions of non-coding RNAs has thrown new light on the regulation of gene expression. At first, the discovery of miRNAs and the subsequent characterization of their functions evidenced their crucial roles in all of the main biological processes: development, differentiation, apoptosis, cell cycle, and diseases including cancer [3, 4]. The involvement of a vast number of miRNAs in the development and progression of ovarian cancer was recently reviewed [5].

More recently, the diversity and number of non-coding RNAs have been extended with the demonstration that at least 75% of the genome is transcribed into non-coding RNAs [6]. Although the functional significance of this entire pool of transcripts is still under debate [7], a vast repertoire of long non-coding RNAs (lncRNAs) has come to light. Unlike siRNAs and miRNAs whose size is usually comprised between 20 to 24 nucleotides, lncRNAs range in size from 200 to more than one hundred thousand nucleotides and bear little or no coding potential. LncRNAs are known to be involved in many biological processes such as imprinting, development and apoptosis and the modes of regulation mediated by these lncRNAs are very diverse [8, 9]. To date, the control of gene expression at the transcriptional level by epigenetic modifications of chromatin is one of the most extensively described [10, 11]. LncRNAs can also play a role in splicing regulation [12], as competing endogenous RNAs [13, 14] or by hosting miRNAs [15, 16]. Owing to their central role in the control of gene expression, lncRNAs are also implicated in cancer [17, 18] including ovarian carcinoma. Although the field of lncRNAs is relatively new, their involvement in ovarian cancer can only be expected to grow. In this review, we briefly present the modes of action of lncRNAs and then focus on the lncRNAs for which a role has been already described in ovarian cancer. In addition, we highlight the importance of the partners with which lncRNAs interact and their associated miRNAs in ovarian carcinoma.

## MODES OF ACTION OF LNCRNAS

### LncRNAs mediate post-translational modifications on histones

The most widely described mode of action of lncRNAs is through interaction *via* their secondary structures with specific protein complexes involved in the epigenetic regulation of gene expression, such as the polycomb repressive complexes PRC1 [19] and PRC2 [20] and the trithorax group protein complex MLL [21]. Many lncRNAs (more than 9 000) have been found to be associated with PRC2 [20], and many accounts describe the interaction of lncRNAs with PRC1, PRC2 or MLL, underlining the importance of their functional relationship [22]. For example, interactions between the lncRNAs HOTAIR, MEG3 or H19 and the protein EZH2 (member of the PRC2 complex), between the lncRNA FAL1 and the protein BMI1 (member of the PRC1 complex) or between the lncRNA HOTTIP and the protein WDR5 [21] (member of the MLL complex) have been reported to be necessary for their functions [23, 24, 20].

The binding of lncRNAs to PRC1, PRC2 or MLL complexes enables targeting to the loci of specific genes. Although the exact mechanisms of such a specific targeting are still not perfectly understood [25], Mondal *et al.* recently demonstrated how MEG3 lncRNA is able to target EZH2 to specific genomic loci. A “GA”-rich sequence in MEG3 allows the recognition and subsequent formation of DNA:DNA:RNA triplex structures in distal regulatory elements for TGFβ pathway genes. The physical association of EZH2 with MEG3 enables the deposition of the H3K27 trimethylation repressive mark at these loci and subsequent inhibition of expression of the TGFβ pathway genes. Indeed, triplex formation might be a general mechanism for the targeting osf specific genes by the lncRNAs involved in chromatin modification [26].

**Figure 1:**
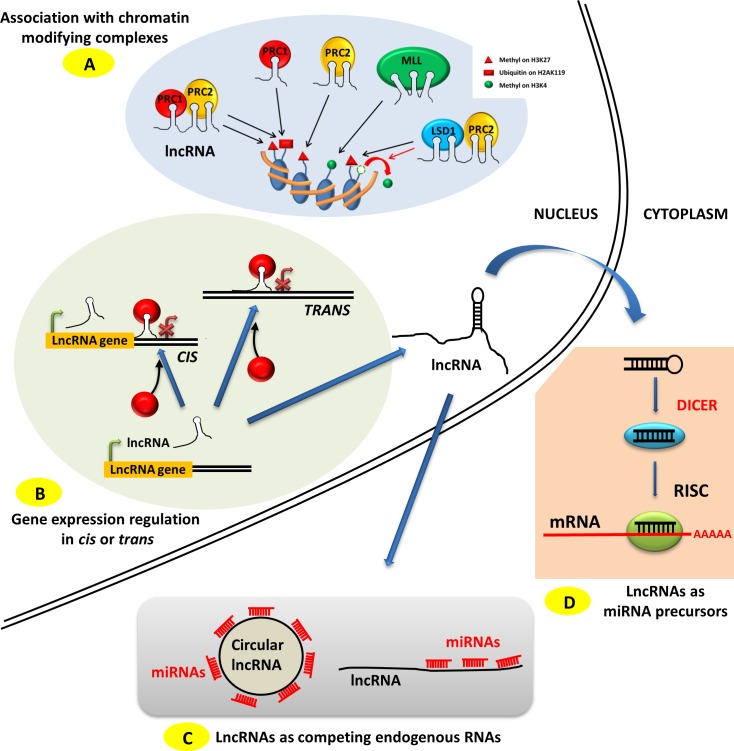
Modes of action of lncRNAs **A.** LncRNAs can associate with various chromatin-modifying complexes such as PRC1, PRC2 and MLL and guide them to specific chromosome locations. PRC1 and PRC2 deposit the transcriptional repressing marks H2AK119Ub1 and H3K27Me3 respectively, and MLL deposits the transcriptional activating mark H3K4Me3. LncRNAs can act as a scaffold by coordinating the action of several chromatin-modifying complexes such as PRC1 and PRC2 or PRC2 and LSD1, the latter removing the transcriptional activating methyl groups on H3K4. **B.** LncRNAs can address chromatin-modifying complexes to specific genomic loci in the vicinity of their transcription site, *i.e.* in *cis*, or at distant sites on the same or different chromosomes, *i.e.* in *trans*. **C.** LncRNAs, either linear or circular, can act as competing endogenous RNA (ceRNAs), sequestrating miRNAs from their mRNA targets. **D.** LncRNAs can act as precursor RNAs for miRNAs.

PRC1, PRC2 or MLL complexes promote post-translational modifications of histone proteins within chromatin (Figure 1A), modulating its compaction and therefore its transcriptional activity for the genes located in the corresponding loci. PRC2 and PRC1 complexes mediate trimethylation on lysine 27 of histone 3 (H3K27Me3) and mono-ubiquitinylation on lysine 119 on histone H2A (H2AK119Ub1) respectively, both modifications triggering transcriptional inhibition [27, 19]. On the other hand, MLL complex trimethylates lysine 4 on histone 3 (H3K4Me3), triggering transcriptional activation [21]. Such chromatin modifications can impact genes either in *cis* (genes whose location is immediately adjacent to the lncRNAs), as observed in gene imprinting mechanisms, or in *trans* (genes located at distant regions on the same or other chromosomes relative to the lncRNAs) (Figure 1B) [28]. Whereas most of the lncRNA have been described to mediate their transcriptional regulation either in *cis* or in *trans*, it has been demonstrated that H19 can act both in *cis* and in *trans* (see [29,30] and below), suggesting that other lncRNAs might also present this characteristic.

LncRNAs can also act as a scaffold to bring together different protein complexes, and therefore activities, in the same location (Figure 1B). This is illustrated by the lncRNA HOTAIR which coordinates PRC2 with the demethylase LSD1 removing methyl groups from H3K4, these two complexes thus cooperating in silencing chromatin at the targeted loci [23]. It has also been shown that the lncRNA ANRIL can bind to both PRC1 and PRC2 [31]. Unlike the interaction with a single protein complex, the extent of these scaffolding functions is still unknown, but several other lncRNAs have been postulated or shown to act as a scaffold [25, 23].

### LncRNAs as competing endogenous RNAs

A number of recent reports have described the existence of competition between transcripts for binding miRNAs, thereby unraveling a new layer of complexity in the regulation of gene expression. One of the first examples of such interaction in mammalian cells was described for PTENP1, a pseudogene of *pten*, whose transcript is able to compete for miRNAs binding with its cognate PTEN transcript and therefore influences its protein expression levels [32]. These miRNAs-sponging transcripts have been termed competing endogenous RNAs (ceRNAs), and it is thought that many lncRNAs, as well as the recently (re)discovered lncRNA class of circular RNAs (circRNAs), participate in ceRNAs-mediated regulatory networks (Figure 1C) [14]. To date, more than 7 000 circRNA have been identified in human cells [33], but the question remains as to the extent to which these circRNAs are able to act as miRNAs sponges. Moreover, the extent of the functionally meaningful regulatory events mediated by these ceRNAs in human cells has recently been challenged [34]. Future studies may provide more detailed information on the real significance of the effects of ceRNA genome-wide, while several examples of functionally relevant endogenous competition for miRNAs mediated by lncRNAs have been described in the literature in the context of ovarian carcinoma [35, 36].

### LncRNAs as precursors for miRNAs

MiRNAs are small non-coding RNAs of approximately 22 nucleotides in length whose main function is the regulation of gene expression at the post-transcriptional level. The miRNA region encompassing nucleotides 2 to 8, also termed “seed”, is able to recognize hundreds of mRNA transcripts through base-pairing in their 3′UTR region, resulting in mRNA destabilization and translational inhibition [37, 38]. MiRNAs originate from long transcripts and are processed by Drosha and DGCR8 into small hairpin-structured double-stranded small RNAs that are exported into the cytoplasmic compartment and eventually loaded into RISC to fulfill their functions. MiRNA genes are located in various genomic contexts, most frequently in intronic regions, but are also found in exonic or intergenic regions [39]. Whereas more than half of the miRNAs genes identified in the human genome are predicted to be processed from coding gene transcripts, 6.4% of the miRNAs (103/1600, according to miRBase release 19) are processed from non-coding transcripts. These 103 miRNAs are hosted within snoRNAs, lncRNAs or other classes of non-coding transcripts, with a strong prevalence for lncRNAs. Indeed 97 miRNAs are hosted within, and processed from, lncRNAs transcripts (Figure 1D). Examples include some well-known lncRNAs, some of which are involved in ovarian cancer biology such as PVT1 and H19 [40]. As a consequence, the effects on gene regulation mediated by lncRNAs hosting miRNAs should be interpreted carefully and by considering the additional effects arising from the miRNA and the lncRNA independently.

## LNCRNAS IN OVARIAN CARCINOMA

### Global transcriptomic analyses identify subsets of lncRNAs whose expression is altered in ovarian carcinoma

With the accumulation of transcriptomic data from high-throughput methods such as microarrays and deep-sequencing, several studies have sought to identify lncRNAs whose expression is deregulated in ovarian carcinomas. In a study integrating the expression profiles of 10 207 lncRNAs from publicly available databases in several cancers [41], it was shown that 1749 lncRNAs displayed an expression specific to one of the four transcriptional subtypes of ovarian cancer (immunoreactive, mesenchymal, proliferative and differentiated) defined by a study on TCGA datasets [42]. Another study reanalyzed TCGA data and showed that 455 lncRNAs were up- or down-regulated in a transcriptional subtype-specific manner [43]. These 455 lncRNAs included NEAT1 and UCA1. NEAT1 is involved in the formation of paraspeckles [44] and was found to be up-regulated in stage III serous ovarian cancer among 113 other genes [45]. Overexpression of UCA1 was shown to increase cisplatin resistance in ovarian cancer cells, possibly through the upregulation of SRPK1 expression [46], a splicing factor whose role in the cisplatin resistance of ovarian carcinoma cells remains controversial [47].

Elsewhere, Perez *et al.* [48] identified a panel of lncRNAs with a differential expression in one ovarian carcinoma patient sample versus normal tissue, but the lncRNAs identified were not functionally characterized. Another study reported 115 lncRNA whose expression is induced in response to estrogen signaling in the SKOV3 cell line and found that among them TC0101441 was involved in migration and invasion in this cell line [49].

Altogether, these observations strongly support a major role for lncRNAs in general in the biology of ovarian carcinoma, although the precise functions of individual lncRNAs in ovarian carcinoma have been less widely explored than in other pathologies.

### Network analysis identifies a subset of lncRNAs possibly involved in ceRNA networks in ovarian cancer

By taking into consideration the ceRNA hypothesis and using published lncRNA-disease associations, available CLIP-Seq data and miRNA-lncRNA interaction databases, Zhou *et al.* [50] attempted to reconstruct disease-lncRNA networks. They designed an algorithm to analyze the possible involvement of lncRNAs within ceRNAs interactions in association with ovarian cancer and ranked them in accordance with the probability of their involvement. Interestingly, MALAT1, MEG3 and HOTAIR were among the top-ranked candidates. The implication of HOTAIR and MEG3 in ovarian cancer is discussed below, whereas MALAT1 has been only partly related to ovarian cancer. Indeed, MALAT1 has been reported to be overexpressed in SKOV3ip ovarian cancer cells, a cell line derived from SKOV3 and harboring a more metastatic phenotype, but the functional implication of MALAT1 overexpression has not yet been investigated [51]. However, MALAT1 has been associated with metastasis and poor prognosis in a number of malignancies [52,53]. In addition, MALAT1 has been implicated in a ceRNA interaction with miR-133 in a myogenesis model [54]. Although this miRNA has not been described in the context of ovarian cancer, its direct targeting of EGFR [55] is an interesting feature, as EGFR downregulation in ovarian cancer cells is known to lead to BIM upregulation and therefore increased apoptosis [56].

### LncRNAs are functionally involved in ovarian carcinoma

Despite the diversity and vast repertoire of lncRNAs, only a handful of them have been shown to be involved functionally in ovarian carcinoma biology or have been proposed as possible biomarkers. Some of these lncRNAs have been studied extensively for their role in several malignancies, whereas the available literature is still rather limited for others. Table 1 combines the lncRNAs that have been studied in the context of ovarian carcinoma, while the available information about their roles and functions is discussed below.

**Table 1:** LncRNAs showing deregulated expression in ovarian tumors

Approved Symbol	Approved Name(Synonyms)	Gene Locus	*In vitro* and *in vivo* observations [ref]	Mode of action	lncRNA expression in patients [ref]	EOC case material (no. of cases)	Normal Counterpart (no. of cases)	Clinical information
**ZNF300P1**	**zinc finger protein 300 pseudogene 1**	5q33,1	**Down-regulation decreases cell-growth and *ex vivo* peritoneal adhesion [57]**	**Not Described**	**Down-regulation [57]**	Clear Cell ovarian tumors patients (8) EOC cell lines (10)	OSE (19)	**N/A**
**AB073614**	**N/A**	3q24	**Down-regulation decreases proliferation, migration, invasion, and promotes apoptosis *in vitro*, and reduces tumor size *in vivo* [58]**	**Not Described**	**Overexpression [58]**	Ovarian tumor patients (75)	Corresponding non-tumor tissue (75)	**Shorter overall survival**
**HOST2**	**Human ovarian cancer specific transcript 2**	10q23.1	**Down-regulation decreases proliferation and migration *in vitro*, and reduces tumor growth *in vivo* [35]**	**miRNAsponging**	**Overexpression [35]**	Ovarian tumor patients (50)	Ovarian benign tumor (30)	**N/A**
**LSINCT5**	**long stress-induced non-coding transcript 5**	5q15.33	**Down-regulation decreases proliferation [60]**	**Not Described**	**Overexpression [60]**	Ovarian tumors patients (15) EOC cell lines (6)	OSE	**N/A**
**FALEC**	**focally amplified long non-coding RNA in epithelial cancer (FAL1, focally amplified lncRNA on chromosome 1)**	1q23.3	**Pro-oncogenic Overexpression suppresses senescence and p21 expression [19]**	**Chromatin modification**	**Overexpression [19]**	EOC tumors patients : early (53) and late (128) stages cell lines (30)	OSE (4)	**Shorter overall survival (late-stage patients)**
**PVT1**	**Pvt 1 oncogene (non-protein coding)**	8q24	**Down-regulation decreases proliferation and promotes apoptosis [62]**	**Myc protein stabilisation**	**Overexpression [62]**	EOC tumor patients (380) EOC cell lines (30)	N/A	**Shorter overall survival**
**XIST**	**X inactive specific transcript(non-protein coding)**	Xq13.2	**Down-regulation decreases resistance to Taxol [70]**	**Chromatin modification**	**Down-regulation [69]**	Paired primary and recurrent ovarian cancer tumors(1 single patient) EOC cell lines (16)	N/A	**Shorter progression-free survival**
**HOTAIR**	**HOX transcript antisense RNA**	12q13.13	**Overexpression increases migration and invasion and promotes EMT [78]**	**Chromatin modification**	**Overexpression [78]**	EOC tumors patients (64) EOC cell lines (5)	OSE (29)	**Shorter overall and disease-free survival (i) advanced FIGO stage (ii) high histological grade (iii) lymph node metastasis**
			**Down-regulation induces cell-cycle arrest *in vitro* and *in vivo* [74]**		**Overexpression [74]**	Serous tumors patients (68) EOC cell lines (6) Xenografts mouse models	OSE (30)	**Shorter overall survival (i) advanced FIGO stage (ii) high histological grade**
			**Overexpression sensitizes to carboplatin effects [75]**		**Overexpression [75]**	Ovarian tumors patients(3 cohorts: 134, 175 and 49)	N/A	**For carboplatin treated patients: Poor outcome Increased risk of relapse**
**HOXA11-AS**	**Homeobox A10 antisense**	7p15,2	**Overexpression reduces proliferation, migration and invasion *in vitro* and reduces tumor size *in vivo* [82]**	**Not Described**	**Down-regulation [82]**	Ovarian tumor patients (18)	Matched ovarian normal tissue (18)	**N/A**
**CDKN2B-AS1**	**CDKN2B antisense RNA 1 (ANRIL, antisense RNA in the INK4 locus)**	9p21.3	**Overexpression increases metastasis [87]**	**Chromatin modification**	**Overexpression [87]**	Serous tumor patients (68) EOC paired parental (2) and metastatic (2) cell lines	OSE (30)	**Shorter overall survival (i) advanced FIGO stage (ii) high histological grade (iii) lymph node metastasis**
**MEG3**	**maternally expressed 3 (non-protein coding)**	14q32.2	**Expression suppresses proliferation and promotes apoptosis [93]**	**Chromatin modification**	**Down-regulation [93]**	EOC tumor patients (20) EOC cell lines (4)	OSE (20)	**N/A**
**H19**	**H19, imprinted maternally expressed transcript (non-protein coding)**	11p15.5	**Hosted miR-675 associated with EMT and chemoresistance [107]**	**Chromatin modification / Hosting miRNA**	**Overexpression [107]**	EOC paired parental (1) and chemoresistant (1) cell lines	N/A	**N/A**
			**Overexpression increases migration and invasion [104]**		**Overexpression [104]**	EOC tumor patients (25) EOC cell lines (2)	N/A	**Correlates with expression of pro-metastatic genes**
			**N/A**		**Overexpression [102]**	Serous tumor patients (41)	OSE (13)	**N/A**

**ZNF300P1** has been described in only one publication so far where it appears to be frequently repressed epigenetically in ovarian cancer cell lines. Down-regulation of this transcript in normal human ovarian surface epithelium (HOSE) cell line was associated with a loss of cell polarity, as well as with increased capacities of adhesion to the peritoneum. This latter finding may be of some importance as spreading in the intraperitoneal cavity and peritoneal carcinomatosis invasion is a very common feature of ovarian cancer [57].

**AB073614** is an lncRNA that was recently found to be overexpressed in ovarian cancer versus normal tissue in publicly available databases. The authors also checked for AB073614 expression in cancer tissue versus normal adjacent tissue in a cohort of 75 ovarian cancer patients and observed that AB073614 high expression was correlated with a shorter 5-year overall survival (17.2 *vs* 30.0 month, *p* = 0.025). *In vivo,* AB073614 downregulation in xenografted mice led to reduced tumor growth and reduced expression of proliferation- and invasion-related proteins (PCNA and MMP2 and MMP9 respectively). *In vitro*, AB073614 was greatly overexpressed in HO-8910 and OVCAR3 cell lines and its downregulation decreased proliferation and induced cell death in them. In addition, PCNA, MMP2 and MMP9 were also decreased *in vitro* in response to AB073614 downregulation, as well as members of key signaling pathways such as the phosphorylated forms of AKT and ERK. Although the mechanisms of action of AB073614 remain to be identified, it appears to be a key regulator of critical processes in ovarian carcinoma cells [58].

**HOST2** is one of the five human ovarian cancer specific transcripts (HOSTs) identified by a SAGE study from a database of 137 SAGE libraries of normal and neoplastic ovarian tissue. Five tags were specifically expressed in ovarian cancer with one of them, HOST2, corresponding to a transcript lacking a coding capacity [59]. HOST2 was confirmed to be overexpressed in ovarian cancer and shown to promote proliferation and migration in cancer cells as well as tumor growth in xenografted mice. The tumorigenic effects of HOST2 are dependent on its ability to act as a molecular sponge for let-7b, from which it is a direct target, thereby impeding the capacity of the latter to down-regulate target genes such as *myc*, *hmga2*, *dicer* and *imp3* [35].

**LSINCT5** has been shown to be overexpressed in many breast and ovarian cancer cell lines and tumor samples. LSICNT5 down-regulation decreases cell proliferation in breast cancer cell lines. A microarray analysis found that LSINCT5 down-regulation triggered the deregulation of 816 genes, of which 95 were deregulated at least 2-fold. Of those, the down-regulation of CXCR4 was postulated by the authors to be responsible for the effects on cell proliferation, but this remained to be demonstrated experimentally [60]. In another study, LSINCT5 was found to be overexpressed in gastric cancer samples versus adjacent normal tissue, and appeared to be an independent predictor of disease-free survival in this pathology [61]. Although these studies provide only limited data regarding ovarian carcinomas, they suggest that LSINCT5 might play a role in these cancers.

**FAL1** was recently identified by Hu *et al.* [19]. This lncRNA is amplified in cancer of various origins. In a cohort of 128 ovarian carcinoma patients, FAL1 was found to be amplified in 38% of cases, this event being associated with a decreased survival. FAL1 associates with BMI1, a member of the PRC1 protein complex, and regulates its stability. Through its association with BMI1, FAL1 regulates the expression of a large set of genes. The oncogenic effects of FAL1 are partly related to its ability to silence transcriptionally the tumor suppressor *p21* (*CDKN1A*), leading to sustained proliferation and reduced senescence. Conversely, down-regulation of FAL1 by siRNA delivery in mice xenografted with ovarian carcinoma cells reduced tumor growth, decreasing cell proliferation and increasing apoptosis.

**PVT1** is an lncRNA located in the 8q24 chromosomal region. This region also harbors *myc,* which is amplified in 45% of ovarian carcinomas. Guan *et al.* [62] reported independent oncogenic roles for MYC and PVT1 in breast and ovarian cancer cell lines, but a recent study by Tseng *et al.* [63] challenged the independence of these roles. They demonstrated that the elevated MYC protein levels in cell lines with 8q24 amplification were dependent on the presence of PVT1 RNA. This co-dependence was further supported by the association of both *pvt1* and *myc* duplication in almost every tumor bearing *myc* amplification. Their analysis included more than 30 000 tumors from the Progenetix dataset and more than 10 000 from TCGA. When all cancers were considered, more than 18% of them presented the duplication of both *pvt1* and *myc*, whereas fewer than 0.5% showed only *myc* or *pvt1* duplicated. In more than 1 000 ovarian cancers from TCGA, more than 45% displayed both *pvt1* and *myc* amplification, with only a few cases (<1%) having only *myc* or *pvt1* duplicated. Interestingly, this study also suggested that the multiple miRNAs encoded in the *pvt1* region did not play a significant role in MYC/PVT1-driven tumorigenesis [64]. In addition, it was recently reported that PVT1 upregulation in response to carboplatin-docetaxel treatment in the 3AO ovarian cancer cell line is a determinant of the induction of p53 and TIMP1 mRNA expression and an associated decrease in cell proliferation [65]. However, because of the frequent missense mutations in the p53 DNA binding domain in ovarian cancer cell lines [66], it would be relevant to check p53 mutational status in 3AO cells to link formally the observed phenotypical effects of PVT1 upregulation with p53 induction. In the same study, 3AO tumor xenografts in mice displayed an induced rate of growth upon PVT1 downregulation. This is in opposition with the study by Tseng *et al.* [63] where *pvt1* inactivation impaired the development of tumor xenografts originating from the colon cancer cell line HCT116. This striking discrepancy might be due to the use of tumor cell lines from diverse origins (ovarian or colon cancer cells) and therefore to the existence of different cellular contexts. This underlines the need for further studies on the exact role of the *myc*/*pvt1* couple in ovarian cancer.

**XIST** is a major player in the complex mechanism leading to heterochromatinization and subsequent transcriptional inactivation of one of the two X chromosomes in women, a phenomenon necessary for dosage compensation. A role for XIST has been described in several malignancies but its effects are dependent on the cancer type. For example, XIST expression is upregulated in glioblastoma tissue and stem cells and its knockdown exerts tumor suppressive functions. These tumor suppressive effects do not rely directly upon the effects of XIST but on miR-152, which is released from its interaction with XIST. Indeed, miR-152 binds to XIST and this miRNA is able to induce anti-tumor effects comparable to XIST downregulation, supporting a ceRNA relationship between miR-152 and XIST in this pathology [67]. Conversely, the deletion of XIST from the blood compartment in mice triggers a myelodysplastic and highly proliferative neoplasm with a very high penetrance (100%) [68]. In ovarian cancer cell lines, XIST expression is decreased and associated with loss of X inactivation [69]. A reduced expression of XIST in patient samples was correlated with a shorter progression-free interval (*r* = 0.653, *P* = 0.001). In the same study, a reduced expression of XIST in several ovarian cancer cell lines was associated with an increased resistance to paclitaxel. However, no modification in the response to cisplatin was observed in cell lines with reduced XIST expression. [70]. Overall, the consequences of XIST expression appear to be context-dependent so further studies are needed to clarify its influence and mechanisms of action.

**HOTAIR** is an lncRNA expressed from the *HOXC* locus on chromosome 12. It has been implicated in the proximal and distal orientation during development by inducing the transcriptional silencing of genes in *trans* in the *HOXD* locus on chromosome 2 [27]. It has been shown that HOTAIR acts as a scaffold lncRNA binding to both PRC2 and LSD1 histone modification complexes through its 5′ and 3′ domains respectively [71]. In breast cancer patients, HOTAIR overexpression is associated with increased metastasis and reduced overall survival. It is also observed in breast cancer cells, where its overexpression alters the H3K27me3 pattern and therefore gene expression. Inversely, HOTAIR depletion inhibits breast cancer cell invasiveness [72]. In human colon cancer (HCC), higher HOTAIR levels are observed in malignant than in non-malignant tissues. High HOTAIR levels are also associated with shorter survival and increased recurrence rates [73]. In a cohort of 68 serous ovarian cancer patients, high HOTAIR expression was associated with advanced FIGO stage (III-IV) and high histological grade (G3) and was found to be an independent prognostic factor of reduced overall survival in a multivariate analysis (36 *vs.* 61 months), similar to FIGO stage and histological grade [74]. However, a more recent study conducted in a larger cohort (134 patients) did not find any association between increased HOTAIR expression and stage or grade, although it was associated with reduced overall survival for carboplatin-treated (but not cisplatin-treated) patients in several cohorts [75]. The first study by Qiu *et.al* [74] included ovarian carcinoma patients with serous subtype, whereas the second study by Teschendorff *et.al* [75] included all subtypes (serous, mucinous, endometrioid, clear cell) as well as fallopian tube and non-classifiable tumors, which could possibly explain this discrepancy. In the A2780cisR cell line, a cisplatin-resistant counterpart of A2780, HOTAIR was expressed 5-fold higher and its downregulation recapitulated cisplatin sensitivity [76]. Similarly, HOTAIR expression led to resistance to cisplatin in several ovarian cancer cell lines through activation of the Wnt/β-catenin pathway [77]. Moreover, HOTAIR down-regulation in xenografted mouse models of ovarian cancer leads to the reduction of tumor weight and the number of peritoneal implants, supporting a role for HOTAIR for *in vivo* cell growth and/or capacity of adhesion to the peritoneum [78]. Interestingly, it has also been shown that HOTAIR is involved in a ceRNA interaction with miR-193a in acute myeloid leukemia, decreasing the ability of miR-193a to interfere with c-KIT expression [79]. This observation could indeed be of interest since miR-193a regulates proliferation and apoptosis in ovarian cancer cells through the post-transcriptional modulation of MCL1 and potentially CCND1, ERBB4 and KRAS [80].

**HOXA11-AS** is an lncRNA located in the *HOXA* locus in chromosome 7 that harbors several coding and non-coding transcripts. HOXA coding genes regulate mullerian duct differentiation and are not expressed in ovarian surface epithelium under normal conditions. However, they are re-expressed in ovarian cancer, including some in a subtype-specific manner. For example, HOXA9 is re-expressed in serous, endometrial and mucinous subtypes, irrespective of grade, whereas HOXA10 is expressed in endometrial and mucinous but not serous subtypes [81]. The 5-prime region of the *HOXA* locus hosts 3 lncRNAs, namely *HOXA10-AS*, *HOXA11-AS* and *HOTTIP*. A recent study looked for variants of these 3 lncRNAs in a cohort of 1947 ovarian cancer patients (1201 of them of serous subtype) and 2009 control cases. No significant variant was identified, although the authors described an A>T variant in *HOXA11-AS* that was marginally associated with reduced ovarian cancer risk (*p* = 0.06). Expression of HOXA11-AS was reduced in ovarian tumor versus matched normal tissue in a cohort of 18 ovarian cancer patients, irrespective of variant status. Plasmid-based expression of HOXA11-AS, either with the “A” or “T” allele, in OVCA-433 and C19 ovarian cancer cell lines reduced proliferation, migration and invasion *in vitro*, as well as tumor burden *in vivo* in a xenograft model with C13 cells. Interestingly, expression of the “T” minor allele increased the inhibitory effects of HOXA11-AS *in vitro* and *in vivo*, in line with a putative reduced risk for ovarian cancer [82]. The authors showed that HOXA11-AS expression has no influence on the expression of its neighboring genes and reported the absence of any predicted miRNA target site, suggesting a possible *trans* regulation of distant genes. However, the mechanisms of the HOXA11-AS effects remain unknown, as well as the reason why the “T” allele displays an increased inhibitory effect. In this regard, the A>T variation does not change the secondary structure of HOXA11-AS. In view of this and given the lack of coding potential for HOXA11-AS, understanding why the “A” and “T” variants show significantly different activities could be of importance for further understanding the modes of action of HOXA11-AS.

**ANRIL** is an lncRNA originating from the 9p21 chromosomal region in the same location as the *p14*, *p15* and *p16* genes, which play a central role in cell cycle regulation, apoptosis and senescence. The ANRIL transcript encompasses *p14*, *p15* and *p16* and is transcribed antisense to them. SNPs in the *anril* locus have been associated with a number of pathologies including cardiovascular diseases, diabetes and endometriosis [83]. ANRIL expression has also been associated with glioma, breast cancer and other malignancies [84] by epigenetically silencing the expression of the p15(INK4b), p14(ARF) and p16(INK4a) locus through its association with PRC1 and PRC2 complexes [31]. The resulting decrease in expression of these tumor suppressor genes reduces senescence [85] and promotes angiogenesis, migration and invasion in breast cancer cells [86]. A recent study demonstrated that ANRIL is an independent prognostic factor in ovarian carcinoma. Overexpression of this lncRNA promotes migration and invasion *in vitro* in ovarian cancer cell lines through the modulation of MET and MMP3, although the authors did not determine whether or not this regulation is dependent on p14, p15 and p16 [87]. It has however been reported that p16 is frequently repressed epigenetically in ovarian cancer, underlining the need for further studies to elucidate the precise roles of ANRIL in ovarian cancer [88].

**MEG3** is an imprinted, maternally expressed lncRNA associated with PRC2, most likely via its EZH2 subunit [20]. It is expressed in humans in several normal tissues and is lost in various tumor types [89], which is associated with poor prognosis in gastric and colorectal cancers [90, 91]. Its ability to induce p53 as well as to inhibit cell proliferation in the absence of p53 underlines its tumor suppressor activity [92]. The *Meg3* promoter was reported to be methylated and MEG3 expression was absent or reduced in ovarian cancer tissues and cell lines. Its re-expression decreased OVCAR3 cell line growth and proliferation [93]. Interestingly, in gastric cancer, methylation of the MEG3 promoter has been linked to miR-148a down-regulation through its ability to target DNMT1 [94], whereas miR-148a reduced expression is associated with a poor prognosis in ovarian cancer patients bearing wild type *brca1/2* [95]. It could therefore be of interest to investigate a possible link between miR-148a and MEG3 expression in ovarian cancer. In addition, miR-26a and MEG3 expression were correlated in TSCC (Tongue Squamous Cell Carcinoma) cells and it has been postulated that this relationship depends on the ability of miR-26a to target DNMT3B in this model and thus prevent *meg3* promoter methylation [96]. However, miR-26a has been shown to promote proliferation and tumorigenesis of ovarian cancer cells through the targeting of ER-α, suggesting a putative different relationship between MEG3 and miR-26a in ovarian cancer that remains to be investigated [97]. Another function of MEG3 has been described in gastric cancer cells, where it shows a competing endogenous activity to miR-181a [98]. This observation is relevant to ovarian cancer, given the role of miR-181a in this pathology where its upregulation is associated with a poor outcome. MiR-181a has been proven to play a critical role in the epithelial-to-mesenchymal transition (EMT) through the direct repression of Smad7 [99]. However, competing endogenous inhibition of miR-181a might not be the sole mechanism for MEG3-repressed EMT, as it was recently demonstrated that MEG3 controls the expression of several genes in the EMT pathway (TGFB2 and SMAD2 among others) at the epigenetic level through an association with EZH2 [26].

**H19** is one of the most widely studied lncRNA and is at the center of a complex regulatory network involving the implication of miRNAs in diverse ways. H19 expression is restricted to fetal tissue and adult muscle under normal conditions [36]. Apart from a role in embryonic development (reviewed by Gabory *et al.* [29]) through effects in *cis* on the *igf2* gene and in *trans* on the imprinted gene network controlling growth in mice [30], H19 has been found to be associated with EZH2 [100] and to be overexpressed in many malignancies [16, 101] including ovarian carcinomas [102]. Alternatively, H19 has been reported to act as a tumor suppressor in mice *in vivo* [103].

One role of H19 is through its ability to sponge the family of tumor suppressor let-7 miRNAs, as shown by Kallen *et al.* in a muscle differentiation model [36]. In ovarian cancer cells, it was shown that H19 overexpression enhances migration and invasion. In this model, the observed effects were mediated, at least in part, by the regulation of some let-7 target genes involved in metastasis [104], suggesting a ceRNA role in this model as well.

However, the involvement of H19 in cancer biology has been mainly described owing to its role as a precursor for miR-675. This miRNA displays an oncogenic role in cancer cell lines from various origins. The tumorigenic functions of miR-675 are mediated, at least in part, thanks to its ability to target directly the tumor suppressor RB in colorectal cancer [16] and hepatocellular carcinoma [105]. MiR-675 also has a role in promoting invasion of glioma cells by targeting cadherin 13 [106]. In ovarian carcinoma, it was shown that a chemoresistant A2780 cell line expressed higher levels of H19 than the sensitive counterpart and this observation was associated with a mesenchymal phenotype in the resistant cell line [107]. In this model, H19 was able to up-regulate SLUG expression, such regulation being dependent on miR-675 and resulting in the suppression of the epithelial marker E-cadherin and inducing EMT. Interestingly, in the same study, a similar mode of action for H19 was shown *in vivo* in mice where H19 overexpression increased the metastatic behavior of lung carcinoma cells [107]. Therefore, more detailed studies on the role of H19/miR-675 might be of interest owing to the chemoresistance and peritoneal invasion usually observed in ovarian carcinoma.

## PROTEINS ASSOCIATED WITH LNCRNAS-INDUCED TRANSCRIPTIONAL SILENCING: BMI1 AND EZH2 ARE INVOLVED IN OVARIAN CARCINOMA BIOLOGY

BMI1 and EZH2, which are members of the PRC1 and PRC2 complexes respectively, and therefore partners in the action of lncRNAs in the control of gene expression, have been shown to be deregulated in ovarian cancer.

Several lines of evidence show that **BMI1** plays an oncogenic role in epithelial malignancies. It is frequently overexpressed in breast, cervical, endometrial and ovarian cancer [108, 109]. In ovarian carcinoma, BMI1 expression correlates with histologic grade and disease stage [110] and is associated with resistance to chemotherapeutic agents such as cisplatin [111]. Interestingly, BMI1 is a direct target of miR-15a and miR-16, both being frequently down-regulated in ovarian cancer. In addition, it was shown that in ovarian carcinoma cell lines and tissue samples, miR-15a and miR-16 levels were inversely correlated with BMI1 expression. [109]. In ovarian cancer cells, the transfection of miR-15a and miR-16 decreased proliferation and clonogenicity. This effect was dependent on the down-regulation of BMI1 and not on that of the anti-apoptotic protein BCL2, another target of miR-15a and miR-16.

Furthermore, an elevated expression of **EZH2**, a methyltransferase subunit of the PRC2 complex, has been shown to be associated with advanced stages of ovarian cancer and to be independently associated with shorter overall survival in ovarian cancer patients [112]. EZH2 knockdown in ovarian cancer cell lines led to reduced cell proliferation and inhibited cell migration and/or invasion *in vitro*. In addition, miR-101 was found to target EZH2 directly in ovarian cancer cell lines. MiR-101 is under-expressed in ovarian cancer tissues and its low expression correlates with FIGO stage but not with tumor grade [113]. *In vivo*, miR-101 restoration leads to the inhibition of growth of ovarian tumor xenografts. In another study using a cohort of patients with ovarian carcinoma, the authors confirmed several observations already made by Semaan *et al*. [113]. They found that miR-101 was down-regulated in advanced ovarian carcinoma FIGO stage and reported a negative correlation between miR-101 expression and histological grade. They confirmed that miR-101 targets EZH2 directly and that their expressions are negatively correlated *in vivo*. Finally, they demonstrated that miR-101 was less expressed in ovarian cancer cell lines resistant to cisplatin than in their sensitive counterparts and that forced miR-101 ectopic expression re-sensitized resistant cells to cisplatin, which is in agreement with the EZH2-induced resistance to cisplatin described in ovarian cancer cells [114]. These findings strongly suggest that the functions of miR-101 in ovarian carcinoma rely on its ability to target EZH2. This is further supported by the observation that miR-101 restoration leads to a decrease in H3K27me3 (a chromatin mark typically deposited by EZH2) at the promoter of the tumor suppressor *p21* and to an increase in *p21* transcription [113], which is in line with the re-expression of p21Waf1/Cip1 triggered by EZH2 downregulation in ovarian cancer cells [115].

Another interesting miRNA in the context of EZH2 is let-7. In ovarian cancer, most let-7 family members appear to be down-regulated (7b [116]; 7d [117]; 7e and 7f [118]; 7i [119]). Although less frequent, up-regulation of certain let-7 family members has also been described, suggesting that let-7 does not always play a tumor suppressor role [120]. A potential relationship between let-7 and EZH2 was suggested in ovarian cancer cell lines when it was shown that let-7e expression was significantly reduced in the cisplatin-resistant cell line A2780/CP compared with parental A2780 cells and that let-7e levels decreased in cells treated with cisplatin. The re-expression of let-7e could both re-sensitize the A2780/CP cell line and lead to a down-regulation of EZH2 [121]. However, the authors did not show whether EZH2 is a direct target of let-7e and a recent study in prostate cancer cell lines suggested that let-7e, unlike other let-7 family members (let-7a, let-7c and let-7b), does not inhibit EZH2 directly [122]. Therefore, the exact link between let-7e and EZH2 expression in ovarian carcinoma cells remains elusive. Recently, a meta-analysis of transcriptomes from high-grade serous ovarian carcinoma patients showed let-7b to be an unfavorable prognostic biomarker that can predict molecular and clinical subclasses of serous ovarian carcinoma patients [123]. Given the ability of let-7b to target EZH2, this observation may appear counter-intuitive. However, in the context of ovarian carcinoma, HOST2 and H19 lncRNAs can act as ceRNA toward let-7b, thereby “sponging” its effects. Further studies on the role of let-7 family miRNAs in ovarian cancer with respect to the transcriptomic background and putative ceRNA networks would throw light on the complex regulatory networks involving this family of miRNAs.

Therefore, the oncogenic roles of both EZH2 and BMI1 and the implication of the lncRNAs/Polycomb axis with miRNAs show that lncRNAs and their functional protein partners are highly involved in the biology of ovarian cancer cells.

## CONCLUDING REMARKS

As was the case with miRNAs a decade ago, lncRNAs now represent a new layer of complexity in the regulation of gene expression. Although only a small subset of lncRNAs has been functionally characterized, it is clear that they are at the center of the most critical physiological and pathological biological processes. In ovarian carcinoma, data already available underline their importance and it can be reasonably expected that future studies will unravel the ever-growing role of lncRNAs in the biology of this malignancy. In addition, increasing evidence of the existence of miRNA-lncRNAs interactions through ceRNAs relationships constitutes another potentially significant regulatory mechanism that needs further exploration and characterization.

From a clinical perspective, lncRNAs expression profiles could improve the stratification of ovarian cancer patients. Alone or in combination with other types of markers (miRNAs, mRNAs, proteins), lncRNAs expression signatures could be used to predict outcome or response to treatment in order to improve the therapeutic care of ovarian carcinoma patients. The recent discovery that miRNAs are present in blood and other body fluids and the potential use of circulating miRNAs as diagnostic or prognostic tools in ovarian carcinoma have opened up new perspectives for the use of easily accessible biomarkers. Regarding lncRNAs, no study has yet reported their presence in serum or plasma from ovarian cancer patients. However, proof of concepts for the meaningful use of lncRNAs present in body fluids has been obtained with the characterization of urinary PCA3 levels as a diagnostic tool in prostate cancer [124], and circulating LIPCAR levels for prognosis after myocardial infarction [125].

Since several lncRNAs are overexpressed (See Table 1 for details) in ovarian cancer and present oncogenic roles, their inhibition might offer interesting perspectives for the treatment of this disease. The most direct approach to achieve lncRNAs inhibition would be the use of RNA interference. However, despite the efforts of the scientific community in the past decade, no RNA interference-based drug has yet obtained approval from the health authorities. In the future, the discovery of ways to deliver siRNAs, miRNAs or their expression vectors safely and efficiently will make the clinical use of RNA interference a reality.

Another strategy to modulate lncRNAs activity, when it relies on chromatin modification mediated by PRC1 and PRC2 complexes, is to use the pharmacological inhibitors of these proteins. Specific inhibitors of EZH2 methyltransferase activity such as GSK126, EPZ005687 and EI1 have been recently developed [126], and it was shown that GSK126 efficiently decreases global H3K27 trimethylation in lymphoma cells and inhibits the growth of diffuse large B-cell lymphoma xenografts in mice, with no apparent toxicity [127]. Therefore, such inhibitors could be used to counter the EZH2-mediated oncogenic effects of overexpressed lncRNAs such as HOTAIR or ANRIL. However, one should keep in mind that the MEG3 tumor suppressive function is also EZH2-mediated. This underlines the need for a selective approach that takes into account the lncRNAs present in a given model and/or tumor before considering EZH2 as a potential therapeutic target in ovarian carcinoma.

Furthermore, the characterization of the genes and pathways modulated by lncRNAs could lead to the identification of potential therapeutic targets for which inhibitors are under development or already available in clinical practice. In addition, the identification of the regulators of lncRNAs expression might provide interesting tools. Using this concept, Mizrahi *et. al.* presented an original strategy in which they used a construct of diphtheria toxin under the control of the H19 promoter. The transfection of this plasmid, BC-819, led to the selective killing of malignant H19-overexpressing cells. Local IP administration of the plasmid vectorized with PEI has reached phase I in ovarian carcinoma patients, with no reported toxicity and encouraging preliminary clinical data [128, 129, 101]. The clinical use of this strategy is even more advanced for bladder cancer with two phase III trials to begin in the first half of 2016.

In summary, the emerging field of lncRNAs has created new opportunities of investigation for a better understanding of ovarian cancer biology. Although limited to a few examples so far, the huge amount of lncRNAs whose roles and functions have yet to be unraveled holds great promise for major improvements in the understanding and future management of this disease.
